# Increased risk of intraoperative and early postoperative periprosthetic femoral fracture with compaction compared with broaching in cementless THA: a single-center study of 6,788 hips

**DOI:** 10.2340/17453674.2024.41341

**Published:** 2024-09-06

**Authors:** Øystein HØVIK, Arild AAMODT, Einar AMLIE, Einar Andreas SIVERTSEN

**Affiliations:** Department of Orthopaedic Surgery, Lovisenberg Diaconal Hospital, Oslo, Norway

## Abstract

**Background and purpose:**

Periprosthetic femoral fracture (PFF) is a significant complication of total hip arthroplasty (THA). Although biomechanical studies have indicated that the technique by which the femoral canal is prepared plays a role, few clinical studies have reported on how this might affect the fracture risk. This study compares the fracture risk between compaction and broaching with toothed instruments in cementless THA.

**Methods:**

Prospectively collected data from the quality register of a high-volume hospital was used. All primary arthroplasties using the Corail stem (DePuy Synthes) were included. All femoral fractures occurring within the first 90 days after the operation were included in the analysis. We determined the relative risk of sustaining PFF with compaction compared with broaching and adjusted for confounders (sex, age group, BMI, and use of a collared stem) using multivariable Poisson regression.

**Results:**

6,788 primary THAs performed between November 2009 and May 2023 were available for analysis. 66% were women and the mean age was 65.0 years. 129 (1.9%) fractures occurred during the first 90 days after the operation, 92 (2.3%) in the compaction group and 37 (1.3%) in the broaching group. The unadjusted relative risk of fracture in the compaction group compared with the broaching group was 1.82 (95% confidence interval [CI] 1.25–2.66), whereas the adjusted relative risk was 1.70 (CI 1.10–2.70).

**Conclusion:**

Compaction was associated with more periprosthetic fractures than broaching (2.3% versus 1.3%) within 90 days after surgery.

Periprosthetic femoral fracture (PFF) is a significant complication of total hip arthroplasty. The reported incidence ranges from 0.7% to 4.9% [1-4]. Several factors influence the risk of PFF, and those factors may be patient-specific or dependent on prosthetic and instrument design.

Risk factors for fracture include female sex, age, preoperative diagnosis, cementless stem, femoral deformities, previous surgery, revision surgery, and the approach to the hip joint [3]. In high-risk patients, it could be advisable to use an anatomical stem and cemented fixation, as this combination seems to be favorable in terms of minimizing the risk of PFF [5,6]. Nevertheless, for a large proportion of patients, cementless stem fixation continues to be used by surgeons [7,8].

Several design elements of cementless stems affect the fracture risk. This includes the shape and surface of the implant, as well as the size and presence of a collar [1,9-11]. A large proportion of fractures occur intraoperatively or in the early postoperative period, before bony ingrowth takes place [2,3,12].

The femoral canal may be prepared either by compaction with smooth tamps of increasing size to compress and preserve the cancellous bone or by broaching with instruments of increasing size with a sharp and toothed surface, thereby removing some of the cancellous bone [13-15]. In experimental canine studies, the compaction technique has shown improved gap-healing and fixation of porous and hydroxyapatite (HA) coated implants [16,17].

Biomechanical studies also indicate that the compaction technique could improve implant fixation [18]. We are, however, not aware of any human clinical studies that support this. Some studies using RSA have found increased migration of the femoral implant in the compaction group or no difference between the groups [14,15,19].

Human cadaver studies have shown an increased risk of femoral fractures using compaction compared with a broaching technique [13,20], but there have been few clinical reports on how the preparation of the femoral canal before implantation might affect the subsequent fracture risk [15,21].

We aimed to compare the fracture risk within the first 90 days following uncemented THA between compaction and broaching with toothed instruments.

## Methods

### Study setting

This study was conducted at a single hospital in a high-volume orthopedic department. It was performed as part of ongoing quality surveillance in the hospital’s orthopedic department and was approved by the hospital’s data protection official. Characteristics of the patients and the surgical procedures are included in the database at the time of surgery. The study was retrospective, and the prospectively collected data from the department’s quality register was used to identify patients sustaining a periprosthetic femoral fracture, either intraoperatively or within 90 days after surgery.

The Corail stem (DePuy Synthes, Warsaw, IN, USA) was introduced as the standard femoral implant in the department for all primary THAs in November 2009 and smooth instruments were used to prepare the femoral canal in all primary procedures until March 23, 2017, when all the instruments were exchanged for toothed broaches. This date serves to separate the THAs using the 2 different techniques.

The study is reported according to STROBE guidelines.

### Technique

**Figure 1:**
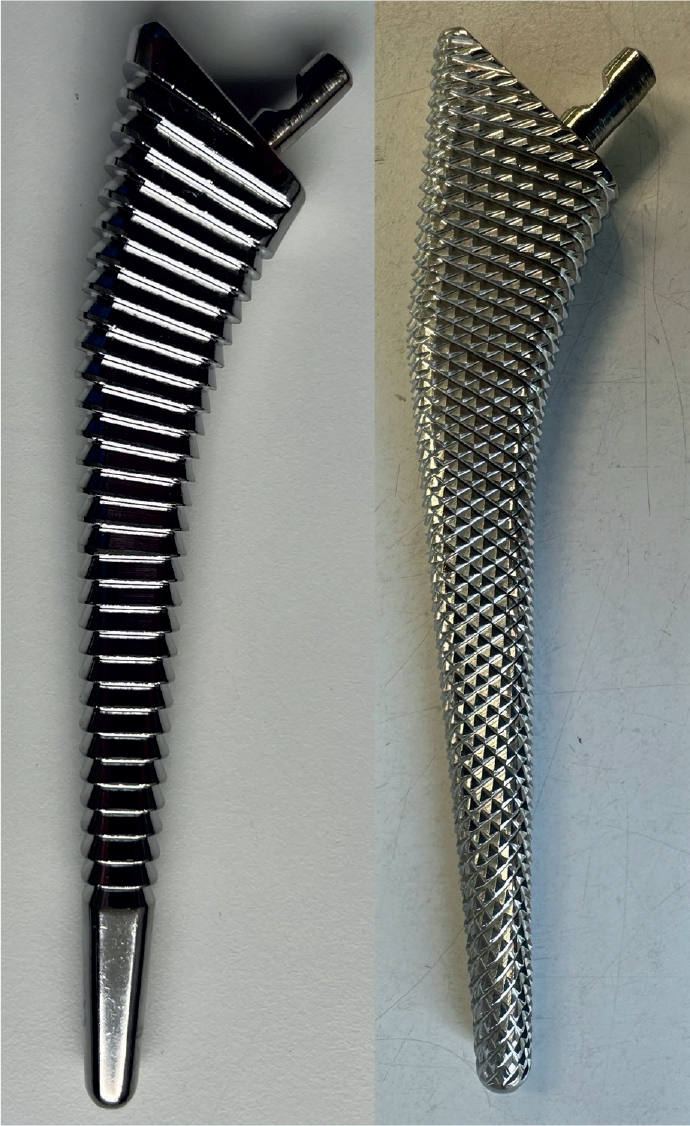
Compaction broach and diamond toothed broach.

In both techniques successively larger broaches are used until stability is reached, confirmed by rotating the broach handle without observing any movement of the broach in the femoral canal. The compaction broach has a surface with smooth horizontal grooves contrary to the other broach, which has a sharp, chipped surface (Figure 1). The size of each broach is the same as the corresponding implant without the HA coating.

The standard acetabular component was initially Reflection (Smith & Nephew Inc, Memphis, TN, USA). This was changed to R3 (Smith & Nephew Inc, Memphis, TN, USA) in October 2015 and again to Trident (Stryker Orthopaedics, Mahwah, NJ, USA) in April 2019.

All procedures were performed using the posterior approach, and the indication for surgery was almost exclusively osteoarthritis, as the department performs only elective surgeries. Thus, neither diagnosis nor approach were relevant as covariates.

### Outcome

The medical records for all patients with fractures were reviewed, and all observed and registered fractures of the femur were included. Based on the medical records and radiographs, the fractures were retrospectively divided into 3 categories based on their anatomic localization: greater trochanter, medial cortex of the femoral neck, or the femur distal to the femoral neck.

Whether operative or conservative treatment was used was also registered, along with reoperations within the first 90 days. The primary endpoint was any observed fracture within 90 days involving any part of the femur.

The 2 techniques were used in 2 different time periods. Risk factors of patient age, sex, American Society of Anesthesiologists (ASA) class, body mass index (BMI), and the use of a collared implant were used in the statistical model. Age was stratified into 4 groups: < 50, 50–64, 65–79, and ≥ 80 years. Body mass index (BMI) was stratified into 3 groups: < 20, 20–29.9, and ≥ 30. The features of the implant were retrieved from the quality register.

### Statistics

Demographic and clinical variables were presented as counts and percentages (%) when categorical and with means and standard deviations (SD) when continuous. Comparisons of proportions were done using a chi-square test and continuous variables were compared using the two-sample t-test. Analysis of factors associated with early periprosthetic femoral fractures was performed using univariable and multivariable Poisson regression. Relative risks (RRs) with 95% confidence intervals (CIs) based on robust standard errors were determined [22]. The RR for femoral canal preparation (compaction vs broaching) was adjusted for potential confounders. Sex, age, BMI, ASA score, and a collared implant were evaluated as potential confounders, and those associated with fracture at a significance level of P < 0.1 in univariable analysis were included as covariates in the multivariable analysis. 3 cases with missing data were excluded from the analysis. Analyses were performed using R version 4.3.1 (R Core team, R Foundation for Statistical Computing, Vienna, Austria, 2023).

### Ethics, registration, data sharing, funding, use of AI, and disclosures

The study was approved by the hospital’s data-protection official. Sharing of raw data from this study is not possible. This is a quality assurance study, based on data from an approved quality register. Thus, no further ethical approval was required. No specific funding was provided for the study. AI has not been used in this work. The authors have no conflicts of interest to declare. Complete disclosure of interest forms according to ICMJE are available on the article page, doi: 10.2340/17453674.2024.41341

## Results

**Figure 2:**
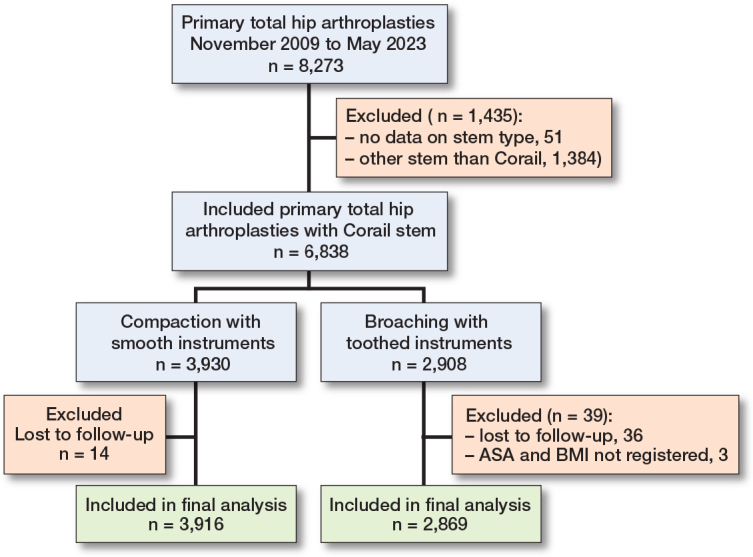
Flowchart of the study cohort.

Data on 8,273 primary THAs performed between November 2009 and May 2023 was collected. After exclusions, 6,788 were available for analysis (Figure 2). 66% were women, and the mean age was 65.0 years (SD 10.0). The patient and procedure characteristics of the total cohort and the 2 study groups are given in Table 1. Patients operated on with compaction were older and more often female. There was also less use of a collared implant in the compaction group. The BMI was also lower in the compaction group.

**Table 1:** Patient and procedural characteristics by type of femoral canal preparation. Values are count (%) unless otherwise specified

Factor	Total cohort n = 6,788	Broaching n = 2,872	Compaction n = 3,916	SMD	P value ^a^
Age ^b^	65.0 (10.0)	63.4 (9.5)	66.2 (10.1)	0.282	< 0.001
Age group				0.350	< 0.001
< 50	534 (7.9)	255 (8.9)	279 (7.1)		
50–64	2,332 (34)	1,112 (39)	1,220 (31)		
65–80	3,559 (52)	1,465 (51)	2,094 (53)		
≥ 80	363 (5.3)	40 (1.4)	323 (8.2)		
Sex				0.076	0.002
Male	2,289 (34)	1,028 (36)	1,261 (32)		
Female	4,499 (66)	1,844 (64)	2,655 (68)		
ASA				0.049	0.3
1	1,081 (16)	471 (16)	610 (16)		
2	4,767 (70)	1,988 (69)	2,779 (71)		
3	925 (14)	407 (14)	518 (13)		
4	12 (0.2)	3 (0.1)	9 (0.2)		
Missing	3	3	0		
BMI ^b^	26.8 (4.5)	27.2 (4.5)	26.5 (4.4)	0.167	< 0.001
Missing	3	3	0		
BMI group				0.139	< 0.001
< 20	175 (2.6)	63 (2.2)	112 (2.9)		
20–29.9	4,977 (73)	2,019 (70)	2,958 (76)		
≥ 30	1,633 (24)	787 (27)	846 (22)		
Missing	3	3	0		
Collared stem	1,998 (29)	1,587 (55)	411 (10)	1.084	< 0.001

a 2 sample t-test; Pearson’s chi-square test.

b Mean (SD).

SMD = standardized mean difference.

The fracture characteristics and treatment types are displayed in Table 2. There were 129 (1.9%) fractures within the first 90 days after the operation. 85 of the fractures occurred intraoperatively. More than half were fractures affecting the medial cortex of the femoral neck (57%, n = 73), followed by fractures distal to the femoral neck and greater trochanter fractures. As most fractures were fractures affecting the medial cortex of the femoral neck discovered during the operation, they were also treated in the same setting, with about half (52%, n = 67) being treated with cerclage around the upper end of the femur. The second most common form of treatment was conservative, with restricted weight-bearing for 6 weeks. 24 (19%) of the patients with fracture underwent reoperation, more often in the compaction group.

**Table 2:** Incidence, location, and treatment of early periprosthetic fractures by type of femoral canal preparation. Values are count (%) unless otherwise specified

Factor	Total cohort n = 6,788	Broaching n = 2,872	Compaction n = 3,916	RR (CI)
Fracture	129 (1.9)	37 (1.3)	92 (2.3)	1.82 (1.25–2.66)
Fracture localization				
Trochanter	18 (0.3)	8 (0.3)	10 (0.3)	0.92 (0.36–2.32)
Medial cortex of the femoral neck	73 (1.1)	16 (0.6)	57 (1.5)	2.61 (1.50–4.54)
Femur distal to the femoral neck	38 (0.6)	13 (0.5)	25 (0.6)	1.41 (0.72–2.75)
Treatment				
Initial surgical treatment	67 (52)	14 (38)	53 (58)	1.52 (0.97–2.38)
Reoperation	24 (19)	5 (14)	19 (21)	1.53 (0.62–3.79)
Nonoperative treatment	38 (29)	18 (49)	20 (22)	0.45 (0.27–0.74)

RR = relative risk, CI = 95% confidence interval.

Fractures occurred at a higher rate in the compaction group than in the broaching group (2.3% vs 1.3%, RR 1.82, CI 1.25–2.66). There was a higher risk of fractures in the age group ≥ 80 years and in the BMI group < 20. Female sex doubled the risk of fracture. There was a tendency towards fewer fractures with use of a collared stem, but it did not reach statistical significance. ASA score was unrelated to the risk of fracture (Table 3). Given these findings, sex, age group, BMI, and use of a collared stem were included in the multivariable analysis as potential confounders. In the multivariable analysis, the adjusted relative risk of sustaining an early PFF when using compaction compared with broaching with toothed instruments was 1.70 (CI 1.10–2.70).

**Table 3:** Results from univariable and multivariable regression analyses with early periprosthetic fracture (PPF) as dependent variable

	Early PPF n (%)	Unadjusted RR (CI)	P value	Adjusted ^a^ RR (CI)	P value
Preparation method			0.001		0.02
Broaching	37 (1.3)	1 Reference		1 Reference	
Compaction	92 (2.3)	1.82 (1.25–2.66)		1.70 (1.10–2.70)	
Age group			0.003		0.04
< 50	5 (0.9)	0.53 (0.21–1.34)		0.56 (0.19–1.30)	
50–64	41 (1.8)	1 Reference		1 Reference	
65–80	66 (1.9)	1.05 (0.72–1.55)		0.97 (0.66–1.44)	
≥ 80	17 (4.7)	2.66 (1.53–4.64)		2.03 (1.11–3.59)	
Sex			< 0.001		0.003
Male	26 (1.1)	1 Reference		1 Reference	
Female	103 (2.3)	2.02 (1.31–3.09)		1.90 (1.24–3.02)	
BMI group			0.003		0.01
< 20	11 (6.3)	3.60 (1.96–6.61)		2.88 (1.45–5.20)	
20–29.9	87 (1.7)	1 Reference		1 Reference	
≥ 30	30 (1.8)	1.05 (0.70–1.59)		1.20 (0.78–1.81)	
Stem			0.08		0.9
No collar	100 (2.1)	1 Reference		1 Reference	
Collar	29 (1.5)	0.70 (0.46–1.05)		1.02 (0.63–1.63)	
ASA score			0.3		
1	14 (1.3)	1 Reference			
2	98 (2.1)	1.59 (0.91–2.77)			
3	17 (1.8)	1.42 (0.70–2.86)			
4	0 (0)	0.00 (0.00–0.00)			

a Adjusted for sex, age group, BMI, and use of a collared stem.

RR = relative risk, CI = 95% confidence interval.

We included interaction terms (covariate*group) in the model to test for a possible modifying effect of selected covariates on the method for preparing the canal. All these tested interaction terms were not statistically significant, with P = 0.3 for sex, P = 0.6 for age group, and P = 0.9 for BMI. However, our data revealed a significant modifying effect of the type of implant (P = 0.01). Thus, we performed stratified analyses for the type of implants. The collared implants had higher risk of fracture when compaction was used compared with broaching (RR 3.37, CI 1.59–7.11) whereas RR was 1.21 (CI 0.75–2.03) for implants without a collar.

## Discussion

We aimed to compare the fracture risk within the first 90 days following uncemented THA between compaction and broaching with toothed instruments. The main finding was that preparing the femoral canal with the use of sharp broaches reduces the risk of intraoperative and early periprosthetic fracture compared with compaction with smooth tamps in cementless Corail stems in THA. The broaching group had a fracture rate within the first 90 days of 1.3% and the compaction group of 2.3%.

In accordance with our study, Hartford et al. reported an increased risk of early fracture rate of 5.8% using compaction compared with 2.8% using broaching [21]. Moreover, Hjorth et al. reported 2 fractures using compaction compared with none using sharp broaching in a radiostereometric analysis (RSA) study of 20 1-stage bilateral primary THA with the Bi-Metric femoral stem [15]. In this study, compaction was used on one side, and broaching with toothed rasps on the other side.

The clinical finding is supported by biomechanical studies indicating that fracture risk could be higher when using compaction. Significantly more fractures developed after compaction than broaching and less force was needed before a fracture occurred. A possible explanation for this could be that the smooth tamp for a given level of force advances more than the toothed broach and thereby generates larger hoop stresses to the femur. In addition, more bone is preserved with compaction than with broaching and this might also lead to increased hoop stresses and fracture [13].

Contrary to Lamb et al., who used early revision for periprosthetic fracture as an end point [11], we found no protection against fracture from an implant collar. As the number of collared implants in the compaction group is very small and as the choice of a collared implant for a normal-offset prosthesis was at the surgeon’s discretion in the first part of the study period, the observation of a larger protective effect of broaching compared with compaction for collared implants compared with collarless must be interpreted with caution.

Although periprosthetic fracture might be a serious complication, the largest group consisted of medial cortical fractures (57%), which were typically dealt with intraoperatively without any serious consequences for the patients. Only 19% of the fractures were reoperated on with either osteosynthesis or a revision stem.

In terms of other risk factors for fracture, our study confirmed the increased risk of periprosthetic fracture among women and patients ≥ 80 years of age [3,12]. We did not find high BMI to be a significant risk factor, but low BMI was, possibly due to its association with comorbidity (e.g., osteoporosis), which may lead to impaired bone quality. We also did not find ASA score to be a significant risk factor for fracture, which contradicts Lamb et al.’s finding that higher ASA score is a risk factor for PFF [11], although their study endpoint was revision surgery, not fracture as used in our study.

### Strengths

In contrast to the other clinical studies that have reported this complication [14,21], our study has a large sample size and 99.3% follow-up rate, which are considerable strengths. In addition, the data are from a single orthopedic department, which reduces sources of variation related to the surgical protocol and patient population but may also limit the generalizability of the findings.

### Limitations

First, the observational design with differences between the 2 groups regarding some of the potential risk factors makes them less comparable. A cemented femoral stem with an anatomical design was introduced in the department as the standard option for patients 80 years and older in August 2013. This age limit was lowered to 75 years in February 2015. As younger patients are more likely to be men, and to have higher BMI, the differences in those parameters could be explained by this change. Second, initially, the lateralized, high-offset variant was the only collared implant in common use. However, collared stems were increasingly used, at the surgeon’s discretion, until they became standard from January 2022. Third, covariables were controlled for in the multivariable regression analysis. However, the different techniques were used in different time periods, which allows for the possibility that factors other than those controlled for could have affected fracture risk. For example, it is possible that awareness of PFF as a significant complication increased over the course of the study period and that this led to more diligent surgery. Fourth, the possible effect of a learning curve could also play a role. When dividing the period until a cemented option was introduced in the department into 3 equally large groups the frequency of fractures in these periods were 1.6%, 2.4%, and 1.8%.

### Conclusion

Compaction was associated with more periprosthetic fractures than broaching (2.3% versus 1.3%) within 90 days after surgery.

In perspective, further investigation is warranted to determine whether different femoral canal preparation techniques impact long-term implant survival.

## Notes

ØH: Conceptualization, data curation, writing – original draft. AA: Methodology, writing – review & editing. EA: data curation, project administration. EAS: Conceptualization, data curation, formal analysis, methodology, software, writing – review & editing.

The authors thank Caryl Gay, MS, PhD, Lovisenberg Diaconal Hospital, Oslo, Norway, for proofreading the manuscript and Kjersti Kaul Jenssen MD, PhD, Lovisenberg Diaconal Hospital, Oslo, Norway for valuable discussions.

Handling co-editors: Keijo Mäkelä and Robin Christensen

*Acta* thanks José Cordero-Ampuero and Stergios Lazarinis for help with peer review of this manuscript.
